# Active and Passive Offline Breaks Differentially Impact the Consolidation of Procedural Motor Memories in Children and Adults

**DOI:** 10.1002/brb3.70138

**Published:** 2024-11-17

**Authors:** D. Voisin, P. Peigneux, C. Urbain

**Affiliations:** ^1^ UR2NF—Neuropsychology and Functional Neuroimaging Research Unit affiliated at CRCN—Centre for Research in Cognition and Neurosciences and UNI—ULB Neuroscience Institute Université Libre de Bruxelles (ULB) Brussels Belgium; ^2^ LN2T—Laboratoire de Neuroanatomie et Neuroimagerie translationnelles affiliated at UNI—ULB Neuroscience Institute, Hôpital Universitaire de Bruxelles (HUB) Université libre de Bruxelles (ULB) Brussels Belgium

## Abstract

**Introduction:**

Short post‐learning breaks, lasting from 5 to 30 min, transiently enhance procedural motor memory performance in adults. However, the impact of activity type (active vs. passive) during the offline break on sequential motor performance remains poorly investigated in children.

**Method:**

This study examined the impact of active versus passive post‐learning breaks on procedural motor memory in 116 healthy participants (58 children, aged 9.03 ± 1.19; 58 adults, aged 22.89 ± 1.77 years). Participants practiced a Finger Tapping Task, reproducing a five‐element keypress sequence as fast and accurately as possible. The task included two sessions (S1 and S2) separated by either a short (30 min) or long (4 h) break. The first 30‐min of the post‐learning break included either a passive (remaining still) or an active (engaging in daily activities) condition.

**Results:**

Repeated‐measures ANOVA revealed significant Session × Age group × Break duration and Session × Break type interaction effects (*p*_s_ < 0.05). Post hoc analyses indicated Session effects in adults after both Break types, but only after short Break duration (S1 < S2, *p* < 0.001; long delay *p* = 0.1). In children, Session effects were observed after both short and long breaks, but only in the active Break type (S1 < S2, *p*_s_ < 0.001; passive condition *p* = 0.1).

**Conclusion:**

These results revealed spontaneous post‐learning motor performance improvements at both short and long delays in children, but only in the active post‐training condition, unlike adults who showed improvements only at short delays, regardless of activity type. This suggests developmental differences in offline conditions (duration and activity) linked to plasticity mechanisms underlying procedural motor memory consolidation.

## Graphical abstract

This study examined the impact of post‐learning breaks on procedural motor memory in adults and children. Results revealed spontaneous post‐learning motor performance improvements at both short and long delays in children, but only in the active post‐training condition, unlike adults who showed improvements only at short delays, regardless of activity type. This suggests developmental differences in offline conditions (duration and activity) linked to plasticity mechanisms underlying procedural motor memory consolidation.

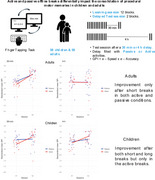

## 1 Introduction

Procedural memory, in particular in its sequential aspect, is instrumental in the acquisition, retention, and execution of automatized behaviors, encompassing skills, habits, and procedures such as cycling, playing an instrument, or writing. Typically, procedural motor memory (PMM) performance (speed and accuracy) markedly and rapidly improves during practice sessions, that is, online. Then, gradual changes in performance spontaneously develop over post‐training offline intervals (i.e., outside task practice), reflecting the ongoing consolidation of newly learned motor skills (Karni et al. 1998; Peigneux et al. 2006; Walker 2005). The offline consolidation of motor memories is a multi‐step, nonlinear dynamic process. In that line, several studies conducted in healthy young adults reported a marked performance improvement when PMM was tested 5–30 min after learning (“boost” phase). In contrast, performance remained similar to initial learning levels when retested 4–5 h later (“silent” phase) (Albouy et al. 2006; Hotermans et al. 2006; Schmitz et al. 2009). Noticeably, improved performance level at the early, transient boost phase was found to reflect subsequent offline performance improvement 48 h later (Albouy et al. 2006; Hotermans et al. 2006), suggesting that immediate post‐training intervals during wakefulness have a functional significance in the development of longer‐term memory consolidation processes (Hotermans et al. 2006). In particular, the boost effect was suggested to be a temporary activated state of motor memory (Hotermans et al. 2006), reflecting the early initiation of PMM consolidation and associated functional brain reorganization processes (for neurophysiological investigations related to the rapid coordination and reorganization of neural networks supporting dynamic PMM and consolidation processes, see Hotermans et al. 2008; Mary et al. 2015, 2017; Peigneux et al. 2006).

The boost effect has been replicated in several studies using various motor sequential tasks (e.g., finger‐tapping, Brawn et al. 2010; Hotermans et al. 2006; Nettersheim et al. 2015; serial reaction time, Schmitz et al. 2009; oculomotor, Albouy et al. 2006) or even mental imagery training tasks (Debarnot, Clerget, and Olivier 2011) in young adult populations. However, the temporal dynamics of offline consolidation processes for procedural motor learning remain poorly understood in children, as do the conditions of a transient boost effect on PMM in children. Still, a large body of evidence suggests that the temporal dynamics of learning and memory consolidation may differ according to the developmental phase. Four‐to‐six‐year‐old and ten‐year‐old children were shown to exhibit offline motor gains 30 min (Wilhelm et al. 2012) and 1 h (Ashtamker and Karni 2013) post‐learning, suggesting a boost effect. Moreover, similar performance improvement was also observed after a 3 h break in 10‐year‐old children (Ashtamker and Karni 2013) and in preschool children after 3–5 h of wakefulness (Desrochers, Kurdziel, and Spencer 2016), contrary to adults in whom no improvement was observed (i.e., silent window; Albouy et al. 2006; Hotermans et al. 2006). Similarly, Adi‐Japha et al. (2019) reported offline gains in PMM performance after a 2 and 4 h break in 5‐to‐6‐year‐old children using a simple grapho‐motor task, while performance stayed at the initial learning level in adults. This suggests that the silent window in adults takes place earlier (< 2 h) than in children and that active plasticity mechanisms at work in the boost phase extend over a longer time scale in children.

Besides the relevance of better understanding the possible differences in the temporal dynamics of PMM processes, a critical point relates to the specific conditions required to observe a boost effect on procedural memories. Indeed, procedural memory consolidation processes benefit from offline post‐learning periods that can include either sleep or wakefulness; the latter can be active or passive. At the behavioral level, resting wakefulness was suggested as equivalent to sleep for procedural memory consolidation (Wamsley 2019, 2022; Wamsley et al. 2023). It was also proposed that reducing external interferences during the post‐learning interval promotes offline consolidation processes (Mednick et al. 2011; Nemeth et al. 2024; Wixted 2004). To the best of our knowledge, only two adult studies compared the impact of an active versus a passive offline post‐learning break on PMM and reported a greater improvement of performance after a passive as compared to an active resting wakefulness condition (Humiston and Wamsley 2018; Wang et al. 2021).

How and whether the type and duration of post‐training intervals similarly impact the evolution of performance in children and adults remain an open question. To address this issue, this study systematically investigated the temporal evolution of motor performance in the boost (30 min) and silent (4 h) post‐learning windows in children as compared to adults, as well as the effect of the active (usual activities) versus passive (quiet rest awake) quality of the post‐learning interval.

## 2 Method

### 2.1 Participants

Sixty‐four healthy children and thirty healthy adults consented to participate (on top of their parents for children) in this study approved by the ULB‐Hospital Erasme Ethics Committee (P2019/663), conducted in accordance with the Declaration of Helsinki. Six children were excluded (three due to a corrupted file, two due to insufficient motor performance, and one due to poor sleep quality [SDSC total score > 67]), as well as two adults due to poor sleep quality (PSQI total score > 7). A final sample of 58 children (35 females; mean age 9.03 ± 1.19, range 7–12 years) and 28 adults (12 females; mean age 22.89 ± 1.77, range 20–26 years) were tested. Participants had no history of psychiatric, neurological, sleep or neurodevelopmental disorders. Sleep quality over the month preceding the experiment was controlled using the Sleep Disturbances Scale for Children (SDSC; Bruni et al. 1996; 36.63 ± 5.97, range 26–57, 1 missing) questionnaire through parental reports and using the Pittsburgh Sleep Quality Index (PSQI; Buysse et al. 1989; 4.86 ± 1.9, range 2–10) for adults. We also included a dataset of 30 healthy adult participants (16 females; mean age 25.8 ± 4.8, range 19–38 years) from a previously published study tested at 30‐min or 4‐h interval in an active (daily activities) break condition (groups 2 and 3 in Hotermans et al. 2006).

### 2.2 Material, Procedure, and Analysis

**FIGURE 1:**
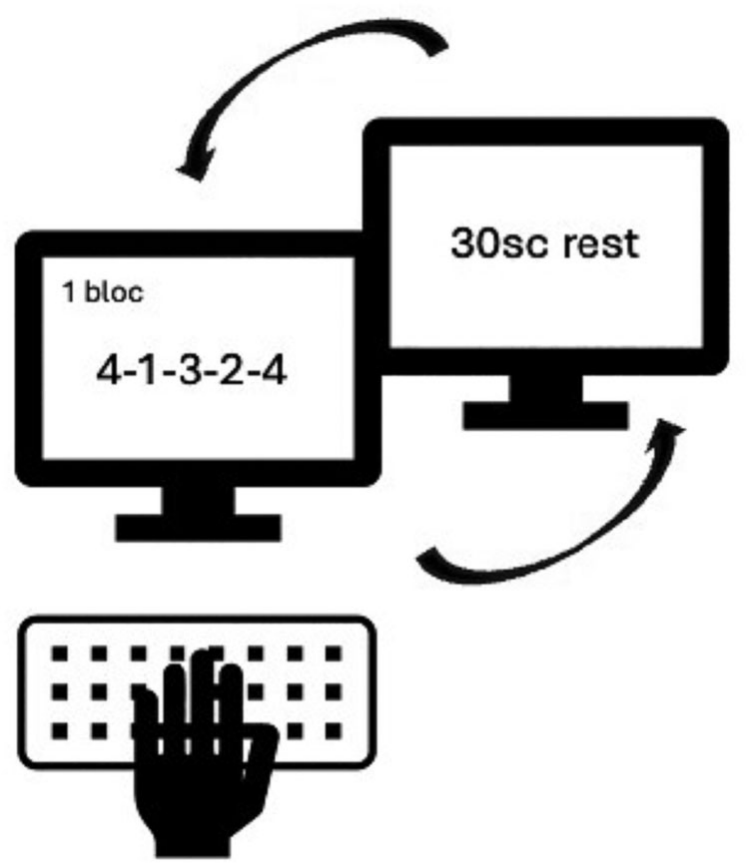
Finger tapping task (FTT). Participants must repeatedly reproduce as quickly and accurately as possible the sequence permanently displayed on the screen. Sequence typing blocks of 30 s are interleaved with 30‐s rest periods.

Participants practiced the motor sequential Finger Tapping Task (FTT; adapted from Karni et al. 1998) during two *Sessions*, a learning session (S1) and a delayed test session (S2). The FTT required participants to repeatedly reproduce as fast and accurately as possible a five‐element sequence of finger movements (4–1–3–2–4; 1 = index to 4 = little finger) for 30 s (i.e., one block) using their non‐dominant hand. The sequence was permanently displayed on the computer screen (Figure 1). The learning session (S1) included twelve 30‐s blocks interleaved with 30‐s rest periods. The test session (S2) took place after a *Break duration* of either 30 min or 4 h and comprised two blocks with the same sequence. The first 30 min of the post‐learning break intervals included either an active or passive *Break type*. The active break type included typical playground activities at school for children and usual daily activities for adults, excluding napping and finger motor practice activities (e.g., keyboard typing). The passive break type involved a wakeful resting interval in a calm environment (i.e., a tipi for children; a sofa for adults), during which participants were asked to remain quiet and still as much as possible without falling asleep. Worth noticing that participant in the 4 h break condition spent the first 30 min either in an active or passive condition, then returned to their daily activities for 3 h and 30 min before being retested. In a between‐subject design, participants were randomly assigned to one of the four conditions (children and adults: 30 min and 4 h passive break condition; children: 30 min and 4 h active break condition). Data taken from the Hotermans et al. (2006) study were in the active break condition (30 min and 4 h).

Motor performance was estimated for each block by computing the Global Performance Index (GPI; Laventure et al. 2016), which accounts for speed‐accuracy trade‐off in performance (Liesefeld and Janczyk 2019). The GPI was computed as “*e‐Speed* × *e*‐*Accuracy*” with *Speed* computed as the duration of a training block (in our case it was fixed at 30‐s) divided by the total number of keypresses in the block and *Accuracy* computed as the difference between the total number of keypresses in a block and the number of correct chunks of three elements produced in the block, divided by the total number of keypresses in the block (see Supporting Information for more details). To control for possible baseline differences in motor speed and/or accuracy between children and adults, analyses were conducted on normalized *Z*‐scored GPI (Lukács and Kemény 2015). Raw speed and accuracy data are provided as Supporting Information. *Z*‐scores GPI were computed at the individual level as the difference between the GPI of a block and the average GPI of all blocks (14 blocks from S1 and S2) divided by the standard deviation of all blocks. Online motor learning was tested using repeated measures ANOVAs on *Z*‐scores GPI across *Blocks* of the learning session (12 blocks of S1). Offline changes between *Sessions* (S1 and S2) were tested using repeated measures ANOVAs contrasting averaged Z‐scores GPI over the last two blocks of S1 (11 and 12) and the two blocks of S2 (13 and 14). *Age group* (children vs. adults), *Break duration* (30 min vs. 4 h) and *Break type* (active vs. passive) were between‐subject factors. Greenhouse–Geisser correction was used when appropriate. Post hoc tests were corrected for multiple comparisons using either Bonferroni or Tukey correction; partial eta‐squared (*η* ^2^ *_p_*) or Cohen's *d* are reported as effect size measures. We also computed Bayesian statistics. Bayesian factor [BF_incl_] values > 3 are strongly supportive of the H1 hypothesis (difference between conditions), BF < 0.33 are strongly in favor of the null hypothesis (no difference between conditions), and 0.33 < BF < 3 are deemed inconclusive (Dienes 2011). Frequentist and Bayesian statistics were performed in JASP version 0.17.3 software.

## 3 Results

### 3.1 Online Learning (S1) Performance

**FIGURE 2:**
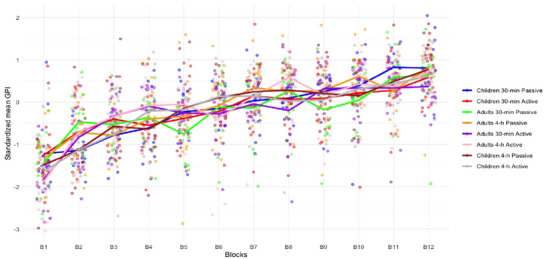
Standardized (*Z*‐scored) mean GPI per block in the learning session (S1: B1–B12) for children and adult participants across the 30 min or 4 h and active or passive delay testing conditions. All groups similarly improved during the learning session.

A repeated measure ANOVA with within‐subject factors *Block* (blocks 1:12) and between‐subject factors *Age group* (children vs. adults), *Break duration* (30 min and 4 h), and *Break type* (active vs. passive) conducted on *Z*‐scored GPI (Figure 2) showed a main *Block* effect *F* (11, 1188) = 87.297, *p *< 0.001, *η* ^2^ *_p_* = 0.447 with progressively increasing GPI across blocks (post‐hoc tests B1, B2, B3, B4, B5, B6 < B10, B11, B12; *p*_s_ < 0.001). All other relevant main and interaction effects were non‐significant (all *p*_s_ > 0.063).

### 3.2 Offline Changes in Performance

**FIGURE 3:**
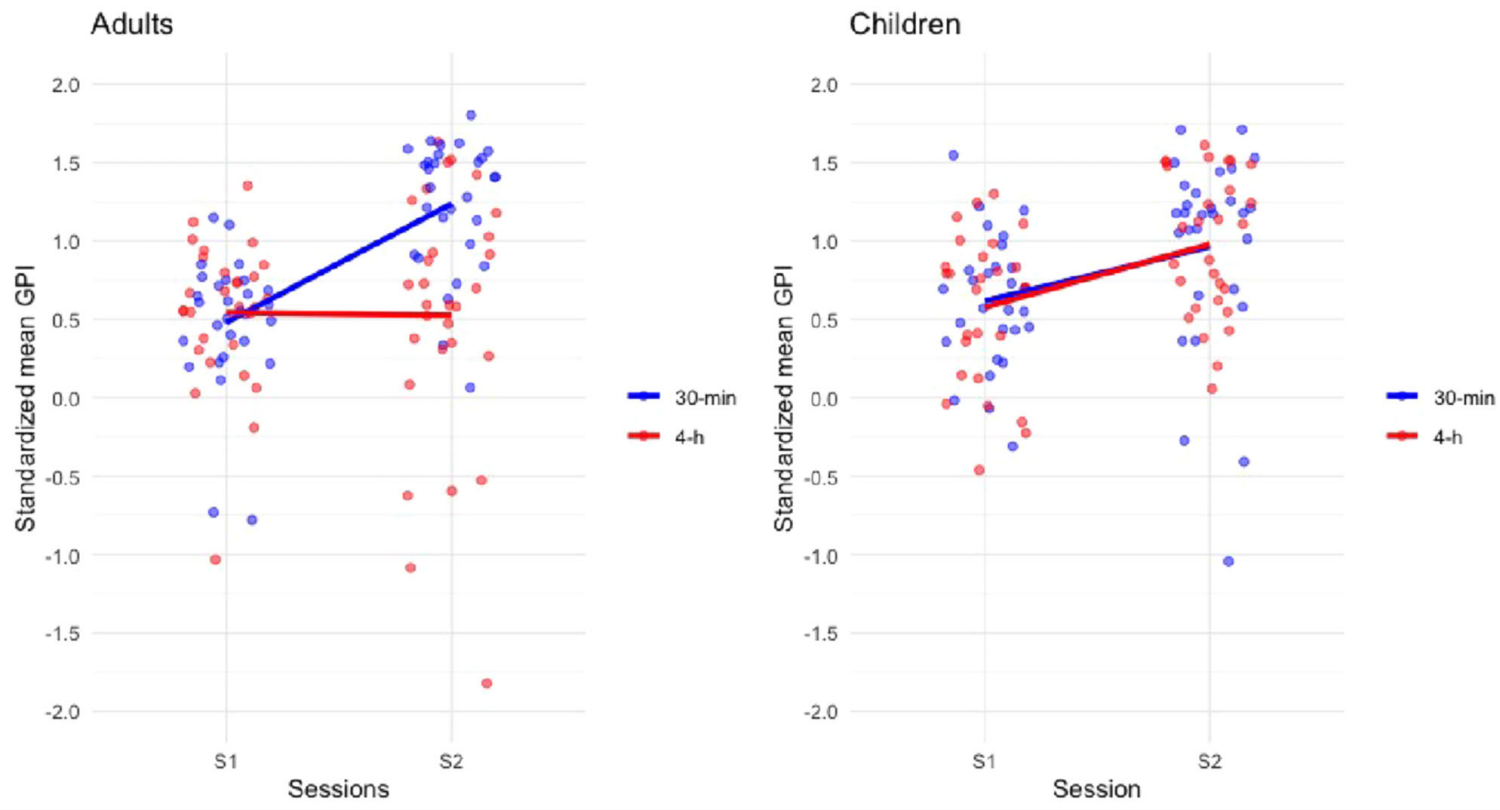
Average *Z*‐scored GPI in the end‐of‐learning (S1: B11–B12) and test (S2: B13–B14) sessions for adults and children participants in the 30 min and 4 h delay testing conditions. Adults improved in the 30‐min break condition only whereas children show a trend towards improvement over the 30‐min and 4‐h break conditions.

A repeated measure ANOVA with within‐subject factors *Session* (S1 vs. S2) and between‐subject factors *Age group* (children vs. adults), *Break duration* (30 min and 4 h), and *Break type* (active vs. passive) conducted on *Z*‐scored GPI disclosed a main *Session* effect *F* (1,108) = 25.692, *p* < 0.001, *η* ^2^ *_p_* = 0.192, BF_incl_ = 121,317.439, with globally improved GPI at the test session (S2), a main *Break duration* effect *F* (1,108) = 6.631, *p* = 0.011, *η* ^2^ *_p_* = 0.058, BF_incl_ = 3.136 with globally higher performance after a 30 min than 4 h break, a *Session* × *Break duration* interaction effect *F* (1,108) = 6.049, *p* = 0.016, *η* ^2^ *_p_* = 0.053, BF_incl_ = 6.868 and a *Session* *×* *Age group* *×* *Break duration* interaction effect *F* (1,108) = 8.298, *p* = 0.005, *η* ^2^ *_p_* = 0.071, BF_incl_ = 5.573 (Figure 3). Post hoc tests showed improved GPI at S2 for adult participants after the 30‐min break (*p* < 0.001, *d* = −1.429) but not after the 4‐h break (*p* = 1.0); however, in children, post hoc tests failed to disclose performance improvement after the 30 min and the 4 h break (*p*_s_ ≥ 0.12), although a trend towards improvement was observed. Finally, the *Session* *×* *Break type* interaction effect was significant *F* (1,108) = 4.386, *p* = 0.039, *η* ^2^ *_p_* = 0.039, BF_incl_ = 0.567, with a significant improvement over sessions in the active (post hoc *p* < 0.001) but not in the passive (post hoc *p* > 0.16) condition. All other relevant main and interaction effects were non‐significant (all *p*_s_ > 0.27).

### 3.3 Exploratory Analyses: Effect of Delay Type in Adults and Children

Given the *Session* *×* *Break type* interaction effect, and despite the non‐significant quadruple *Session* *×* *Age group* *×* *Break duration* *×* *Break type* interaction effect (*p* > 0.9), we decided to conduct exploratory analyses on the effect of *Break type* on performance, separately in children and adults.

**FIGURE 4:**
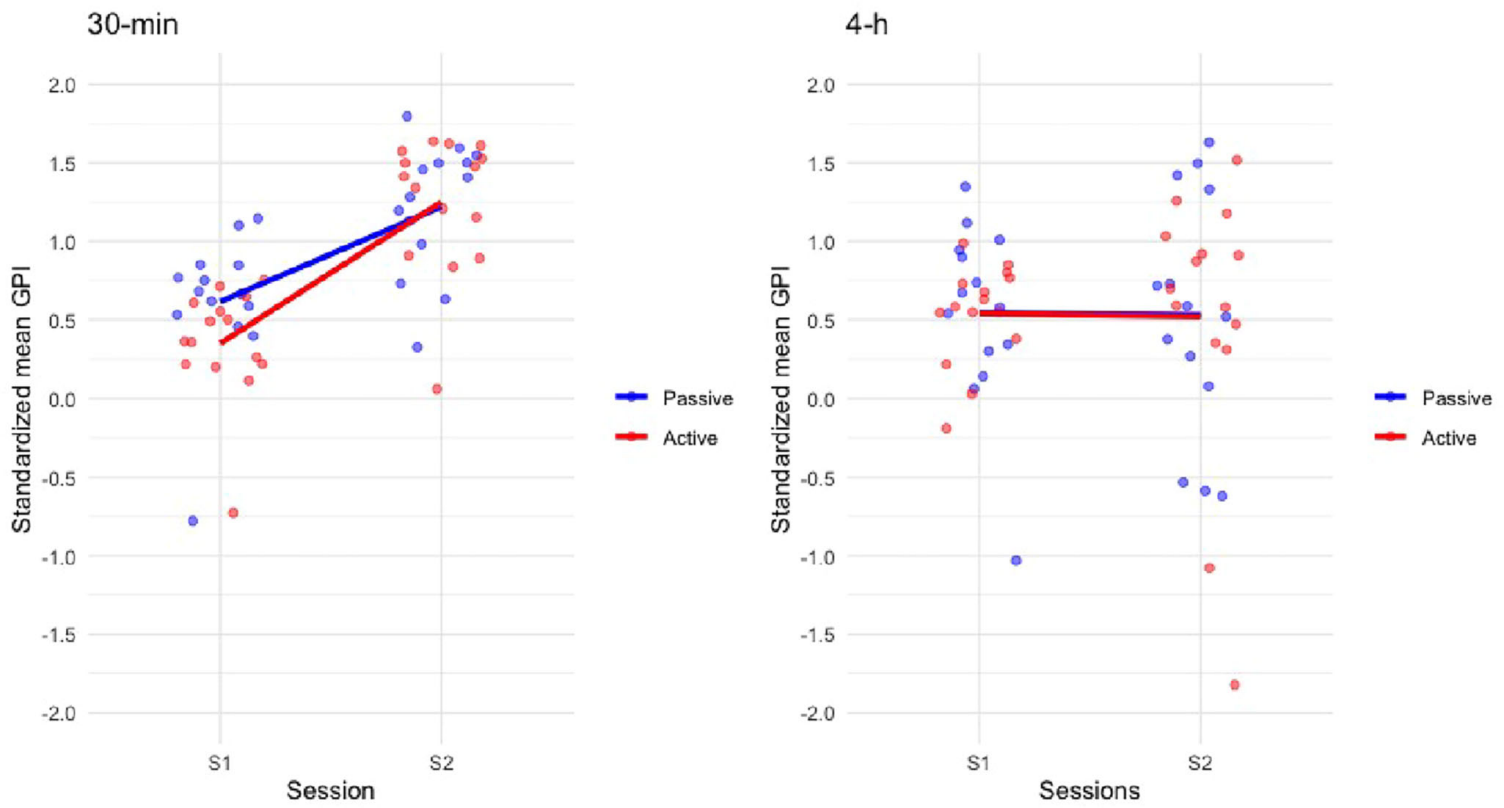
Adults. Average *Z*‐scored GPI in the end of learning (S1: B11–B12) and test (S2: B13–B14) sessions for adults in the passive or active delay testing conditions after a 30‐min or 4‐h delay duration. Adults improved performance over the 30‐min but not 4‐h interval irrespective of the active or passive break condition.

In adults, a repeated measure ANOVA with within‐subject factors *Session* (S1 vs. S2) and between‐subject factors *Break type* (active vs. passive) and *Break duration* (30 min vs. 4 h) conducted on *Z*‐scored GPI (Figure 4) disclosed a main *Session* effect *F* (1,54) = 13.025, *p* = 0.001, *η* ^2^ *_p_* = 0.194, BF_incl_ = 6012.91 with improved GPI at test S2, a main *Break duration* effect *F* (1,54) = 9.348, *p* = 0.003, *η* ^2^ *_p_* = 0.148, BF_incl_ = 566.067 with globally higher performance after 30 min than 4 h break, and a *Session* *×* *Break duration* interaction effect *F* (1,54) = 14.426, *p* < 0.001, *η* ^2^ *_p_* = 0.211, BF_incl_ = 521.408. Post hoc tests showed improved GPI at the S2 test for adult participants in the 30 min (*p* < 0.001, *d* = −1.344) break condition but not in the 4 h (*p* = 1.0) break. Most importantly, the *Session* *×* *Break type* was not significant *F* (1,54) = 0.510, *p* > 0.478, *η* ^2^ *_p_* = 0.009, BF_incl_ = 0.22, suggesting that *Break duration* effects are irrespective of the active or passive delay condition in adult participants. All other relevant main and interaction effects were non‐significant (all *p*_s_ > 0.46).

**FIGURE 5:**
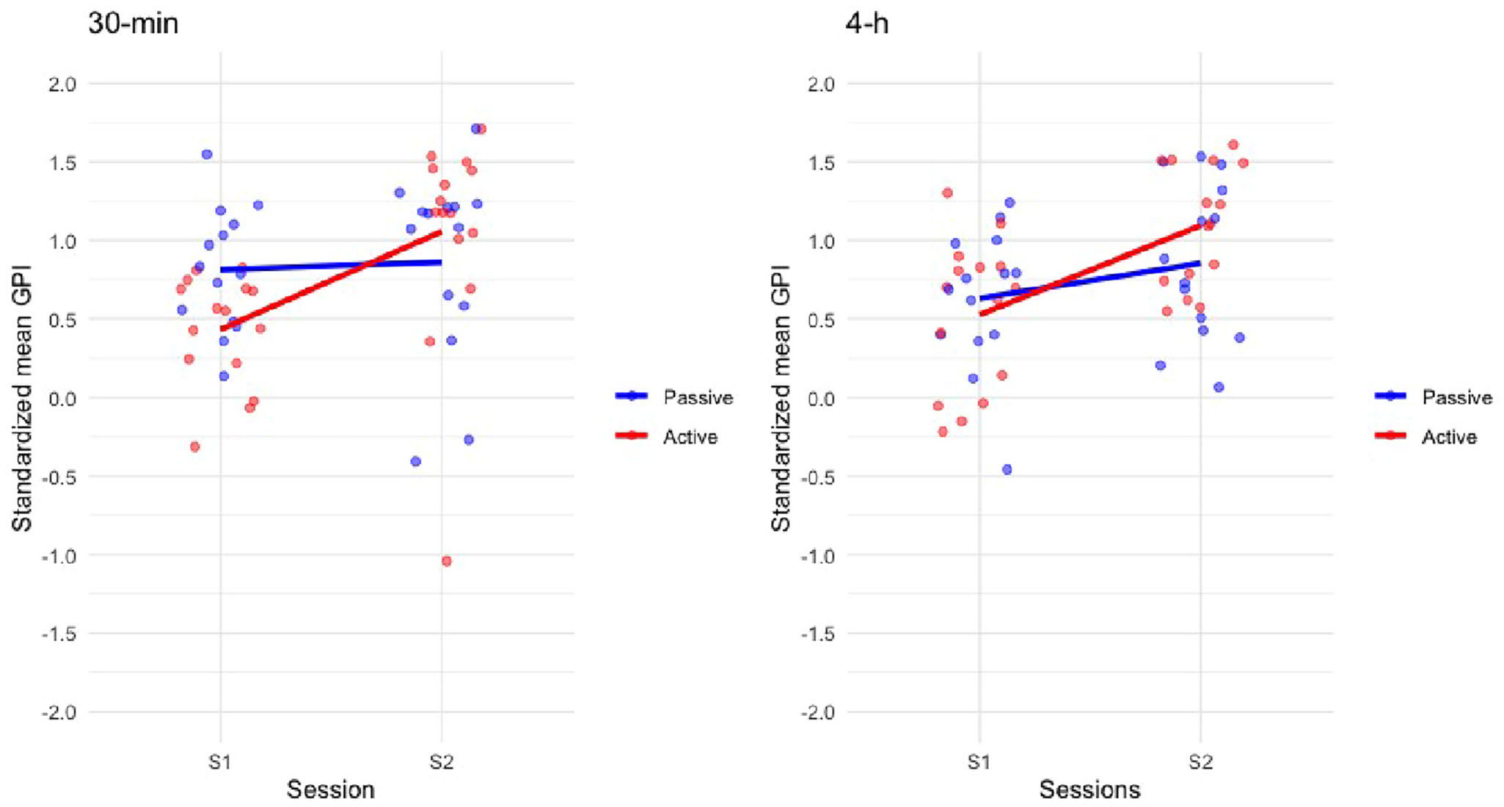
Children. Average *Z*‐scored GPI in the end‐of‐learning (S1: B11–B12) and test (S2: B13–B14) sessions for children in the passive or active delay testing conditions after a 30‐min or 4‐h delay duration. Children improved performance both over the 30‐min and 4‐h interval but only in the active break condition.

In children, a repeated measure ANOVA with within‐subject factors *Session* (S1 vs. S2) and between‐subject factors *Break type* (active vs. passive) and *Break duration* (30 min vs. 4 h) conducted on *Z*‐scored GPI (Figure 5) showed a main *Session* effect *F*(1,54) = 12.671, *p* < 0.001, *η* ^2^ *_p_* = 0.190, BF_incl_ = 349.239 with improved GPI at test S2, and a *Session* *×* *Break type* interaction effect *F*(1,54) = 5.011, *p* = 0.029, *η^2^_p_* = 0.085, BF_incl_ = 2.336. Post hoc tests revealed improved GPI at S2 test for participants in the active delay testing condition (*p* < 0.001, *d* = −1.213) but not in the passive (*p* = 1.0) delay testing condition. Here, the *Session* *×* *Break duration* was not significant (*F*(1,54) = 0.088, *p* > 0.768, *η* ^2^ *_p_* = 0.002, BF_incl_ = 0.138, suggesting that *Break type* effects are irrespective of the 30 min or 4 h break condition in children, at variance with adults. All other relevant main and interaction effects were non‐significant (all *p*_s_ > 0.30).

## 4 Discussion

In this study, we investigated possible developmental specificities regarding the temporal evolution of PMM consolidation processes in school‐age children and young adults and the differential impact of the post‐learning break duration (30 min vs. 4 h) and activity type (active vs. passive). Our results highlight distinctive post‐learning temporal dynamics in the evolution of motor performance in children and adults, with a replication in adults of the transient improvement after 30 min (boost windows) but not 4 h (silent window) delay, whereas children showed improvement both after a 30‐min and a 4‐h break. More specifically, we found a differential effect of the type of activity allowed during the offline break window, with significant performance improvements in children observed only in the active, but not in the passive post‐training condition, and equivalent performance changes in adults irrespective of the type of activity during the delay.

As expected, we replicated a robust, transient boost effect in motor performance in the adult population (Albouy et al. 2006; Brawn et al. 2010; Hotermans et al. 2006; Nettersheim et al. 2015; Schmitz et al. 2009). However, in adults, the type of post‐training activity (active or passive) did not modulate performance, although reducing external interferences during the post‐learning interval (i.e. passive condition) could have promoted better offline consolidation (Mednick et al. 2011; Nemeth et al. 2024; Wixted 2004). To the best of our knowledge, only two studies investigated the impact of active versus passive wakefulness delays on procedural motor performance in adults (Humiston and Wamsley 2018; Wang et al. 2021). Contrary to our results, both found a higher improvement after, respectively, a 15 min (Humiston and Wamsley 2018) or 30 min (Wang et al. 2021) passive, eye‐closed resting period compared to an active (i.e., distractor task) resting period. This positive effect of passive wakefulness was not observed in the group of participants tested after a 4‐h resting period (Humiston and Wamsley 2018). Worth noticing, that in our study, participants enrolled in the passive condition stayed in a calm environment but were not told to keep their eyes closed. Consequently, one potential explanation for these divergent findings could be that our passive condition was actually “less quiet” than a fully passive eyes‐closed condition, a hypothesis that needs further research to be validated.

In our study, where we specifically targeted the boost and the silent windows (based on adult studies), school‐age children in the active, daily activity condition showed offline improvement in PMM both after a 30‐min break and after the 4‐h break. These results are in line with previous studies using motor sequence learning or simple grapho‐motor tasks, in whom performance improvement was found when children were retested after 30 min (Wilhelm et al. 2012), after 1 or 3 h (Ashtamker and Karni 2013), or after 2 or 4 h (Adi‐Japha et al. 2019), but did not specifically investigate the boost effect. These results strikingly contrast those observed in adults, in whom performance improved after a 30‐min but not 4‐h break. Here we show that post‐learning temporal dynamics of PMM performance markedly differed between children and adults.

Moreover, our results highlighting divergent performance evolution in children and adults demonstrate developmental differences in the offline evolution of PMM and modulation with age of the boost effect, which has been considered a predictor of subsequent overnight offline performance improvement in adults (i.e., consolidation 48 h later; Albouy et al. 2006; Hotermans et al. 2006). These results thus do not support the recently proposed hypothesis of an age‐invariant model of procedural memory consolidation (Tóth‐Fáber et al. 2023).

Our results suggest a shorter, more temporally restricted boost window in adults (< 2 h) than in children, and, on the other hand, that active plasticity mechanisms at work in the boost phase expand on a longer time scale in children. This is in line with recent studies showing larger performance gains over macro‐ and micro‐offline intervals in children, suggesting a developmental advantage in the offline processing of recently practiced sequences of movement (Du et al. 2017; Van Roy et al. 2024).

Still, the presence of a silent window remains to be demonstrated in children. Future studies should retest children after longer (> 5 h) offline breaks to ascertain that performance is back to end‐of‐learning levels (i.e., silent period) like in adults after a few hours. The mechanisms underlying these developmental differences in the post‐learning, offline evolution in performance are nowadays unclear. A tentative explanation could be based on the different learning strategies between children and adult populations. Indeed, some studies suggest that children exhibit a developmental advantage in the offline processing of motor sequences, whereas adults would favor online learning strategies (Du et al. 2016; Du et al. 2017; Van Roy et al. 2024). At the neurophysiological level, the impact of the offline condition on motor performance in children also deserves further investigation. For instance, mu re‐synchronization power (occurring after mu desynchronization) was found to increase with age and interpreted as indicative of reduced cortical inhibition, which might enhance plasticity and motor learning in children (Démas et al. 2020; Gaetz et al. 2010; Kurz et al. 2016; Trevarrow et al. 2019). And on the other hand, the boost effect was found to be disrupted in healthy aging and linked to offline changes in mu‐beta modulation, suggesting reduced plasticity mechanisms (Mary et al. 2015, 2017).

Again, at variance with adults, we found performance improvement in children only when they benefitted from an active post‐learning period, but not after a passive, quiet, controlled resting session. In the latter case, performance remained at end‐of‐learning levels regardless of the timing of the retest (30 min or 4 h), that is, a performance similar to the silent window in adults. Our active condition is ecological in that it reflects the daily life breaks that occur between learning sessions in school‐age children. In our experiment, children in the active condition were typically allowed break time in the playground, whereas those in the passive condition were kept in a classroom and allowed calm activities. Besides a potentially positive effect of being in an interactive social group in the active condition (e.g., children in elementary school would mostly socialize on the playground; Blatchford, Baines, and Pellegrini 2003), another contributing factor might be differences in physical activity during the post‐learning period. Indeed, children spend on average more than 50% of their time in the playground having physical activities (Massey, Ku, and Stellino 2018) that were shown beneficial for cognitive functioning and supportive of learning at school (Etnier and Landers 1997). Also, children who spend more time engaged in physical activity during the day (either in the playground, in physical education, or after school) perform better academically than those who spend more time in instruction (Hillman et al. 2014; Waite‐Stupiansky and Findlay 2002). Hence, these results may be in line with the literature showing the benefits of active, undirected breaks on children's social and cognitive skills (Jarrett 2002; Pellegrini, Huberty, and Jones 1995) and academic success (Health et al. 2013).

In the present study, we included a dataset from the original study by Hotermans et al. (2006) in which adults were tested after 30 min or 4 h in an active delay condition (excluding motor typing activities). It may be argued that 20‐year‐old adults tested in 2006, that is, 20 years ago, have less developed typing skills than the current young adult generation, who comparatively spend more time on phones and computers. However, baseline differences in performance were controlled using Z‐scored data, and offline changes in motor performance were similar between these groups in an active condition (groups 2 and 3 in Hotermans et al. 2006) and the adult groups tested in a passive post‐training condition, suggesting that pre‐learning skill expertise was not a confounding factor in our results.

## 5 Conclusion

To sum up, our study highlights spontaneous post‐learning, offline, motor performance improvements at both short and long delays in children but only in an active post‐training condition, at variance with adults who improved performance only at short delays both in the active and passive conditions. This suggests the presence of developmental differences in the offline conditions (i.e., duration and activity) associated with plasticity mechanisms subtending PMM consolidation.

## Author Contributions

**D. Voisin**: data curation, formal analysis, methodology, software, visualization, writing—original draft, writing—review and editing. **P. Peigneux**: Conceptualization, formal analysis, funding acquisition, methodology, project administration, resources, supervision, validation, writing—review and editing. **C. Urbain**: Conceptualization, funding acquisition, methodology, project administration, resources, supervision, validation, writing—review and editing.

### Peer Review

The peer review history for this article is available at https://publons.com/publon/10.1002/brb3.70138.

## Supporting information




**Figure 1**: Mean speed per block in the learning session (S1: B1–B12) for children and adult participants in the 30 min or 4 h and passive or active delay testing conditions.
**Figure 2**: Mean accuracy in % per block in the learning session (S1: B1–B12) for children and adult participants in the 30 min or 4 h and passive or active delay testing conditions.

## Data Availability

The data that support the findings of this study are available from the corresponding author.
