# Supplementary material for: Active and Passive Offline Breaks Differentially Impact the Consolidation of Procedural Motor Memories in Children and Adults
Source: Brain Behav. 2024 Nov 17;14(11):e70138. doi: 10.1002/brb3.70138 (PMC11570425; doi:10.1002/brb3.70138)
Supplement: Supplementary file 1 — Figure 1: Mean speed per block in the learning session (S1: B1–B12) for children and adult participants in the 30 min or 4 h and passive or active delay testing conditions. Figure 2: Mean accuracy in % per block in the learning session (S1: B1–B12) for children and adult participants in the 30 min or 4 h and passive or active delay testing conditions. [file BRB3-14-e70138-s001.docx]

# Supplementary Information Material.

In this study, the Global Performance Index (GPI) was computed as “*e* - *Speed* x *e* – *Accuracy*” with *Speed* as the duration of a training block (in our case it was fixed at 30 seconds) divided by the total number of keypresses in the block and *Accuracy* as the difference between the total number of keypresses in a block and the number of correct chunks of 3 elements produced in the block, divided by the total number of keypresses in the block. *e* is the mathematical constant, also known as the Euler’s number, and is defined as the base of the natural logarithm (~2.71828).

Here, we report raw Speed and Accuracy for each block of the learning sessions (S1: 1-12) and for each group, as classically computed by Hotermans et al. (2006). That is Speed as the number of chunks of 3-element belonging to the sequence produced for each 30-second block and Accuracy as the percentage of correctly generated chunks of 3-element belonging to the sequence out of the total number of chunks of 3-element generated for each 30-second block.


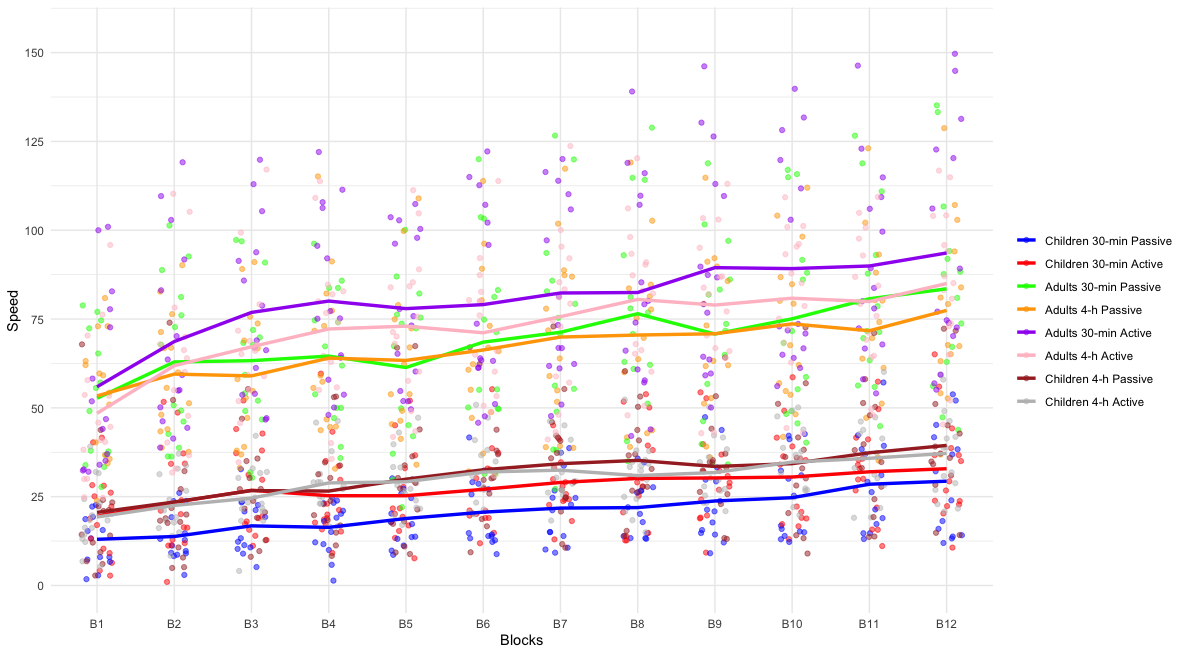


**Figure 1:** Mean Speed per block in the learning session (S1: B1-B12) for children and adult participants in the 30 min or 4 h and Passive or Active delay testing conditions.


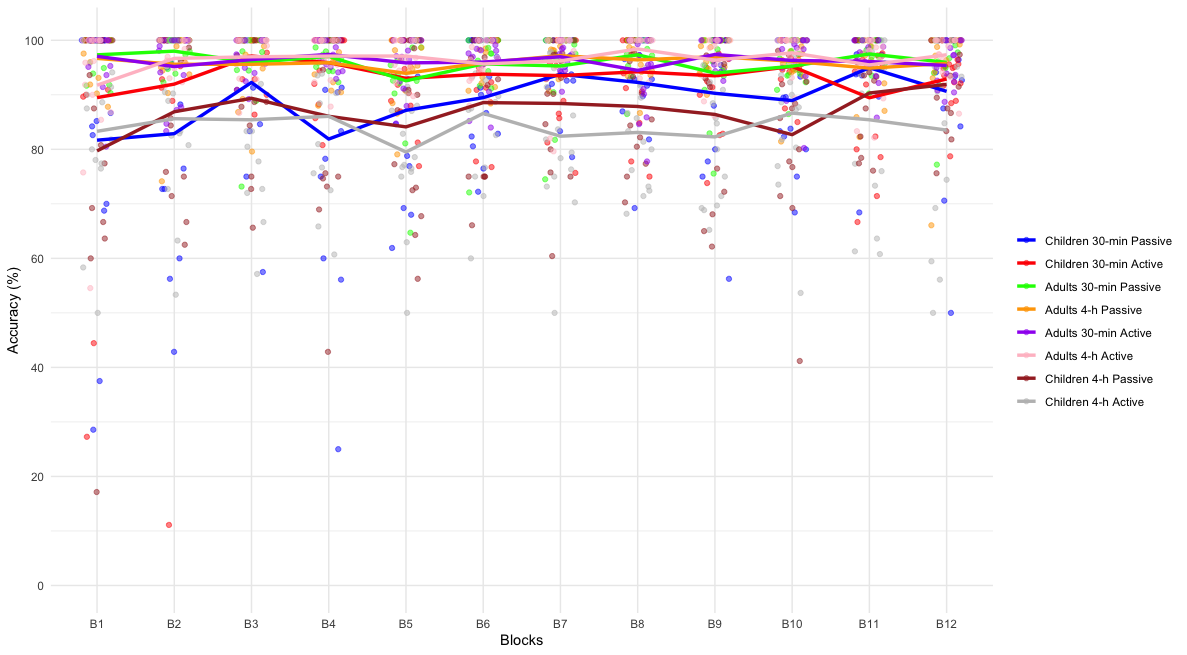


**Figure 2:** Mean Accuracy in % per block in the learning session (S1: B1-B12) for children and adult participants in the 30 min or 4 h and Passive or Active delay testing conditions.
